# Environmental Gradient Favours Functionally Diverse Macrobenthic Community in a Placer Rich Tropical Bay

**DOI:** 10.1155/2013/750580

**Published:** 2013-06-17

**Authors:** S. K. Sivadas, B. S. Ingole, C. E. G. Fernandes

**Affiliations:** Biological Oceanography Division, CSIR-National Institute of Oceanography, Dona Paula, Goa 403004, India

## Abstract

The present paper examines the functional diversity-environment relation in a placer rich tropical bay. Understanding the environmental variables that determine the biodiversity pattern will help in the effective conservation plans of coastal habitat. However, few studies have been carried out on the biodiversity-environment relation from the diverse tropical coastal ecosystem. The geographic location of Kalbadevi Bay along the west coast of India provides an opportunity to study the functional diversity pattern of macrofauna along an environmental gradient. Additionally, the area is also a potential placer mining site. Seasonal sampling was carried out for macrofauna and environmental variables. Macrofaunal functional diversity showed significant temporal variation related to the environmental parameters. The most important environmental variables were organic matter and sediment texture. Filter feeders dominated during postmonsoon which is a period when the water column is enriched with sinking detritus. The deposit feeders which rapidly ingest the settled detritus and also transport it to deeper sediment for the subsurface deposit feeders dominated during premonsoon. Abundance of carnivores was high during premonsoon, a response to increase in food in terms of deposit feeders. The result thus indicates that the temporal environmental variation influenced the macrofaunal functional diversity pattern in the Kalbadevi Bay.

## 1. Introduction

Marine coastal ecosystem is unique due to the dynamic interaction with atmospheric and terrestrial system. This interaction results in high biological productivity and resources of important economic value. Many coastal waters associated with adjacent ecosystem act as a source for placer minerals washed from upstream. Placer deposits are accumulation of heavy minerals formed by concentration of resistant heavy mineral particles of high-specific gravity by the action of waves, currents, and winds [1]. Several of the world's important minerals have been obtained from placer minerals [2]. Further, such coastal areas are of particular interest, as they are subjected to extremely variable environmental condition due to fluctuations in river inputs, which are in turn controlled by climatic conditions. These environmental gradients play an important role in structuring the benthic community of the region and in turn the ecosystem functioning.

Considerable studies have been carried out to understand the role of environmental factors in structuring the macrobenthic community in tropical coastal habitats [3, 4]. However, the majority of the studies were based on species diversity, and very few studies have focused on the functional diversity, especially in the tropical developing countries [5]. Functional diversity is one of the appropriate tools for measuring the ecosystem functioning as it reduces the complex natural ecosystem to comprehensible level [6]. Moreover, functional diversity reflects the diversity of morphological, physiological, and ecological traits within the communities and explains the ecosystem functioning in a better way than other classical taxonomic diversity measures [7]. Further, the ability of ecosystem to resist or return to their former state following disturbance may depend upon the given levels of functional diversity. A highly functional diverse ecosystem will adapt quickly to disturbance compared to impoverished ones. Further, a species with particular functional trait will exist in a region based on the environment which helps it to coexist in a community [8]. Therefore, functional diversity is increasingly used to understand the biodiversity-environment relation and biodiversity-ecosystem functioning and to decipher the effect of anthropogenic activities on ecosystem [9]. 

One of the greatest challenges when determining the impact from mining or any disturbance is the inability to separate man-made impacts from natural changes [10]. An understanding of the natural variability is a prerequisite when establishing reference conditions to avoid misclassification of any system. Hence, the knowledge of reference condition is extremely useful for effective conservation plans [11, 12]. The Kalbadevi Bay along the west coast of India has been identified as a potential site for placer mining. Moreover, the bay is invaded by creeks fringed with mangrove vegetation on the Northern and Southern ends. In tropical rivers and estuaries, the major riverine runoff occurs during the monsoon period which brings about drastic changes in the physicochemical environment of the system [13]. The monsoon period is characterized by maximum precipitation while post- and premonsoon are marked by minimum rainfall to dry period. The post- and premonsoon are relatively more stable periods. Therefore, such dynamic coastal habitat provides an opportunity to understand the relationships linking community parameters, species biology, and ecosystem functioning [14]. Therefore, we tested the hypothesis that the macrofaunal diversity in the Kalbadevi Bay will vary along a spatio-temporal scale, and the differences are related to the environmental variation.

The Kalbadevi Bay, invaded by creeks and influenced by the annual tropical rainfall, provides an opportunity to understand the role of environment in influencing the functional diversity pattern of macrobenthos. Hence, in the present study, we use functional traits along with structural diversity to describe the natural variation in the macrofaunal community from a placer rich tropical bay. The main aim of this study is to determine the role of environmental variation in structuring the functional diversity of macrobenthic community of a placer rich tropical bay.

## 2. Materials and Methods

### 2.1. Study Area and Sampling

**Figure 1:**
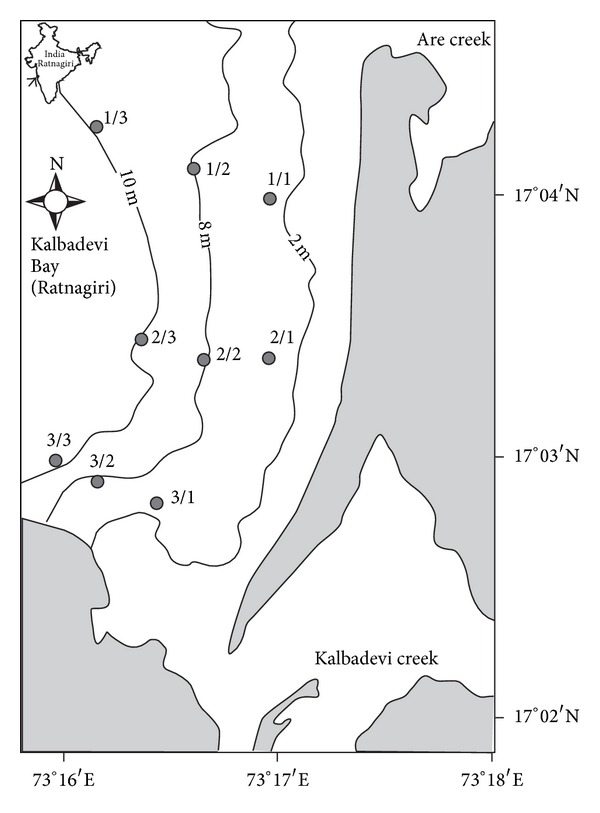
Location of sampling transects (1–3) in Kalbadevi Bay, India.

Sampling was conducted at Kalbadevi Bay (lat. from 17°02′68′′ to 17°04′07′′N; long. from 73°16′93′′ to 73°17′32′′E; Figure 1). The bay is ~5 km long and 3 km wide with Are creek in the north and Kalbadevi creek in the south. The Kalbadevi Bay is considered as one of the important placer deposits along the west coast of India [15, 16]. The occurrence of the deposits is reported to extend from 2 to 5 km offshore and from ~9 to 12 m in depth. The mineral concentration varies from 1 to 91%, including 1–52% of ilmenite with the estimated reserves of 2 million metric ton. 

Samples were collected from three transects and three depths (2, 5, and 8 m) during postmonsoon (January 2006). Transect 1 was located at the north side of the bay, towards the Are creek; transect 2 was located ~1.5 km from transect 1; transect 3 was located south of the bay and ~1.5 km from transect 2. However, the sampling during the next two seasons, that is, premonsoon (May 2006), and monsoon (September 2006) was restricted to two transects (2 and 3) due to the rough weather conditions. The samples were labeled as follows: postmonsoon—Post M; premonsoon—Pre M and monsoon—Mon. Numerical notations were assigned to transect and depth and were indicated together as transect/depth, wherein the first number indicated transect (1, 2 or 3) and second number indicated the water depth (2 m—1, 5 m—2, and 8 m—3).

Sediment from each location was collected in triplicate using a van Veen grab (0.04 m^2^). The samples for macrofauna were sieved through 500 *μ*m sieve. The materials retained on the sieve were preserved in neutralized 10% seawater formalin-rose Bengal. In the laboratory, the samples were carefully washed again, sorted, and preserved in 5% buffered formalin. The faunae were then counted (ind m^−2^) and identified up to the lowest possible taxa under a stereomicroscope. Biomass (g m^−2^) was estimated by wet weight method. 

Sediment sample was collected for biochemical parameters such as carbohydrate (CHO), protein (PRT), lipid (LIP), organic carbon (OC), chlorophyll *a* (Sed Chl* a*), and phaeopigment (Sed Phaeo) and stored at 5°C until further analysis in the laboratory. Chl *a* and Phaeo were extracted overnight in 10 mL of 90% acetone under cold and dark conditions, and the supernatant was estimated using Fluorometer [17]. OC was estimated by digesting 1 g of sediment sample in chromic acid, and the digested matter was titrated with ferrous ammonium sulphate using phenanthroline as an indicator [18]. PRT concentration in the sediment was estimated by Folin-Ciocalteu reagent [19] using albumin as standard. CHO was determined with d-Glucose as standard [20]. LIP concentration was assayed by chloroform and methanol (5 : 10) after ultrasonication in Milli-Q water to improve extraction [21, 22]. All analyses were run in triplicates (100 mg dry weight each). Labile organic matter (LOM) was calculated as the sum of PRT, CHO, and LIP. Quality of LOM is expressed as PRT/CHO ratio. Sediment samples for grain size analysis were prewashed and treated with 10% sodium hexametaphosphate for dispersion before being subjected to analysis [23].

Water samples were collected with 5 L Niskin bottles from 1 m below the surface and 2 m above the sea bed. For Chlorophyll *a* (W Chl *a*) and Phaeophytin (W Phaeo) concentrations in water, 1 L of water was filtered through GF/F filters (pore size 0.45 *μ*m) and extracted in 10 mL of 90% acetone [17]. W Chl *a* and W Phaeo were calculated by integrating the depth values. The data for dissolved oxygen (DO) and nutrients like nitrate (NO_3_), nitrite (NO_2_), phosphate (PO_4_), and silicate (SiO_4_) were obtained from published report [24].

### 2.2. Functional Group

Macrofaunae were divided into functional groups based upon their trophic guilds, mobility, and habit type. Trophic groups used in this study were surface-deposit feeder (SDF), subsurface-deposit feeder (SSDF), filter feeder (FF), grazer (GR), omnivore (O), and carnivore (C). Mobility categories included: mobile (M), discretely mobile (D), and sessile (S). Five categories of habit type were classified free living, that is, living on surface or actively burrowing (F), tubiculous (T), burrow-dwelling (B), attached to hard substrate (A), and anchored in the sediment (U). The data for the functional traits were collected from various published literature and reports [25, 26].

### 2.3. Statistical Analysis

Two-way ANOVA and Students Newman-Keuls post hoc (SNK test) analysis were performed to find out the significance of spatial and temporal variation of environmental and biological parameters. The biological data were log (*x* + 1) transformed, and the *α* (0.05) value was modified after Bonferroni correction to *α*′ (0.0013) [27]. To compare macrofaunal communities between transect and seasons, analysis of similarity (ANOSIM) was also applied. The environmental variables of the study area were first characterized by principal-components analysis (PCA). The PCA was performed on the normalized environmental data. The PCA provides information on the most meaningful parameters and helps to summarize the statistical correlation among environmental variables with minimum loss of original information [28]. The spatio-temporal pattern of macrofaunal community was analyzed by hierarchical agglomerative clustering with group-average linking on the Bray-Curtis coefficient [29] on the log transformed abundance data of species and functional groups. Nonmetric multidimensional scaling (nMDS) was also performed using the Bray-Curtis similarity matrix to produce an ordination plot. A similarity profile (SIMPROF) test was carried out for detecting statistically significant cluster [30, 31]. Similarity percentage programme (SIMPER) was then used to identify the species contributing to the similarity within the group (indicator species) and those species responsible for dissimilarity between groups (discriminating species). Indicator and discriminating species were identified based on average similarity (dissimilarity)/standard deviation ratio and percentage similarity/dissimilarity contribution. A Species with large similarity/dissimilarity percentage and small SD value will have large similarity (dissimilarity)/standard deviation ratio and hence are considered to be significant indicator/discriminating species [29]. A reliable indicator species will have high abundance across the stations (high percentage similarity) and consistent abundance (high SIM/SD ratio). While a reliable discriminating species will have the highest abundance (highest percentage dissimilarity) in one group, but will be rare in the other group. The BIOta ENVironmental matching (BIO-ENV) procedure was employed on the similarity matrix based on abundance data to relate macrofaunal assemblages (abundance, dominant species, feeding, mobility, and habit) to environmental parameters [29]. The analyses were carried using Primer 6 and Statistica 10 software packages. Sediment composition, mean grain size (D_50_), and sorting data were analyzed using GRADISTAT 5 software [32].

## 3. Results

### 3.1. Environmental Parameters

The environmental variables are given in Table 1. The environment in the Kalbadevi Bay showed a temporal variation. PCA analysis on environmental parameters resulted in three components that explained 61% variability (Table 2). The highest positive values on PC1 were found for sand, D_50_, whereas PRT, OC, mud, and W Chl *a* had the highest negative loading. The second PC was influenced by Sed Chl *a* and PO_4_ (negative loading), while NO_2_ showed positive loading. CHO, Sed Phaeo, and sorting had the highest negative loading, while PO_4_ had positive loading on the third axis. PO_4_ concentration, which had the highest negative loading on PC 2 and 3, showed significant (*F* = 7.82; *P* = 0.006) seasonal variation (Table 2). NO_2_ showed the highest values during Post M and lowest in Pre M at all the stations. W Chl *a* showed significant seasonal variation (*F* = 15.18; *P* = 0.0005) with the highest values during Pre M (Table 3).

Sand was dominant (>95%) at all stations, while silt fraction ranged from 0.2 to 2%. During Mon, there was a decrease in silt content. Sand and mud content showed significant spatio-temporal variation (Table 3). The D_50_ value ranged from 88 to 173 *μ*m during the study with significantly high values during Mon (Table 3). Sorting (**σ**_I_) ranged from 0.3 to 0.6, 0.3 to 0.7, and 0.3 to 0.6 for Post M, Pre M, and Mon, respectively. Overall, the sediment grain size was moderately to well sorted. Seasonally, high Sed Chl *a* values were recorded during Pre M (1.27 ± 0.21 *μ*g g^−1^) and low values (0.4 ± 0.1 *μ*g g^−1^) during Mon. Sed Phaeo showed a similar pattern with high values during the Pre M (0.54 ± 0.4 *μ*g g^−1^) and low during Mon (0.19 ± 0.17 *μ*g g^−1^). Sed Phaeo: Sed Chl *a* ratio was the highest during Post M at 3.0 and the lowest during Pre M and Mon at 0.6. OC ranged from 0.1% (Stn 3/1 premonsoon) to 0.5% (Stn 3/3 Post M). Seasonally, average OC value was significantly high during Post M (0.27%) and Pre M (0.24%). OC and Sed Chl *a* showed strong seasonality (*F* = 6.44; *P* = 0.012). However, no significant variation was observed among transects (Table 3). LIP ranged from 19 (Stn 2/1 Mon) to 264 *μ*g g^−1^ (Stn 3/1 Post M). CHO ranged from 132 *μ*g g^−1^ at Stn 1/2 (Post M) to 583 *μ*g g^−1^ (Stn 1/3 Post M). PRT ranged from 204 (Stn 3/1 Mon) to 4640 *μ*g g^−1^ (Stn 2/1 Mon). PRT formed the dominant constituent of labile organic matter (avg. 66%) followed by CHO (21%) and LIP (13%). The PRT contribution to LOM was the highest during premonsoon season (74%) whereas CHO and LIP were the highest during monsoon. None of the main effect or interaction effect (ANOVA analyses) of the biochemical components (PTR, CHO, and LIP) was significant. 

### 3.2. Macrobenthic Community

A total of 116 taxa belonging to 11 phyla were collected from Kalbadevi Bay during the present study (the appendix). Polychaete dominated the community with 81% of the total abundance. Crustaceans at 10% were the second dominant group. Average faunal abundance was higher in Post M (20,625 ± 10,461 ind m^−2^), and significantly lower values were recorded during Mon (4,420 ± 4,912 ind m^−2^). ANOVA and post hoc test showed significant (*F* = 27.86; *P* < 0.001) seasonal variation (Table 4). However, no significant variation was observed between transects and stations. Abundance was low at Stn 3/2 during Mon (850 ind m^−2^) and maximum at Stn 2/3 (33,825 ind m^−2^) during Post M. Two-way ANOSIM result also confirmed that macrofaunal communities showed significant seasonal variation (*R* = 0.72, *P* = 0.1%).

**Figure 2:**
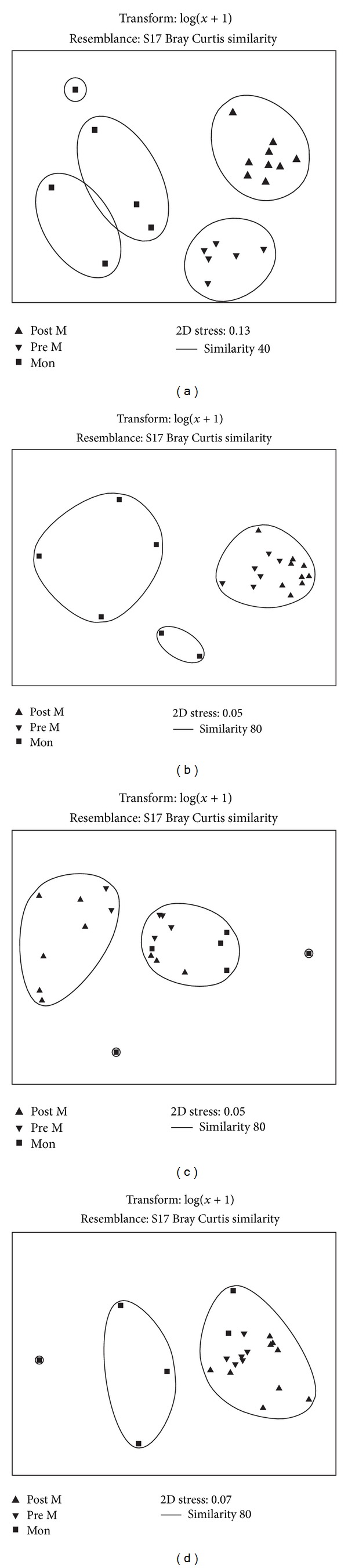
nMDS based on log transformed (log *x* + 1) data of (a) total macrofaunal abundance, (b) feeding guilds, (c) habit, and (d) mobility.

The nMDS based on macrofaunal abundance clearly demarcated the community based on seasons (Figure 2(a)). Post M community was 58% similar to 10 taxa contributing to 52% overall average similarity. Among the 10 indicator taxa, unidentified Sabellidae, *Mediomastus* sp., *Synchelidium* sp., *Ampelisca* sp., Nemertea, *Phyllodoce* sp., and *Minuspio cirrifera* were consistently abundant with high SIM/SD ratio (>5). In general, unidentified Sabellidae species abundance ranged from 2,400 to 16,925 ind m^−2^ and showed significant seasonal variation (Table 4). The species showed 99% reduction during Pre M and was totally absent during Mon. Highest abundance of *Mediomastus* sp. was recorded at Stn 2/3 (15,075 ind m^−2^) and lowest at Stn 3/2 (250 ind m^−2^). This species did not show any significant spatio-temporal variation (*P* > 0.05). *Synchelidium* sp. and *Ampelisca* sp. showed significant seasonal variation with the highest values recorded during Post M (Table 4).

**Figure 3:**
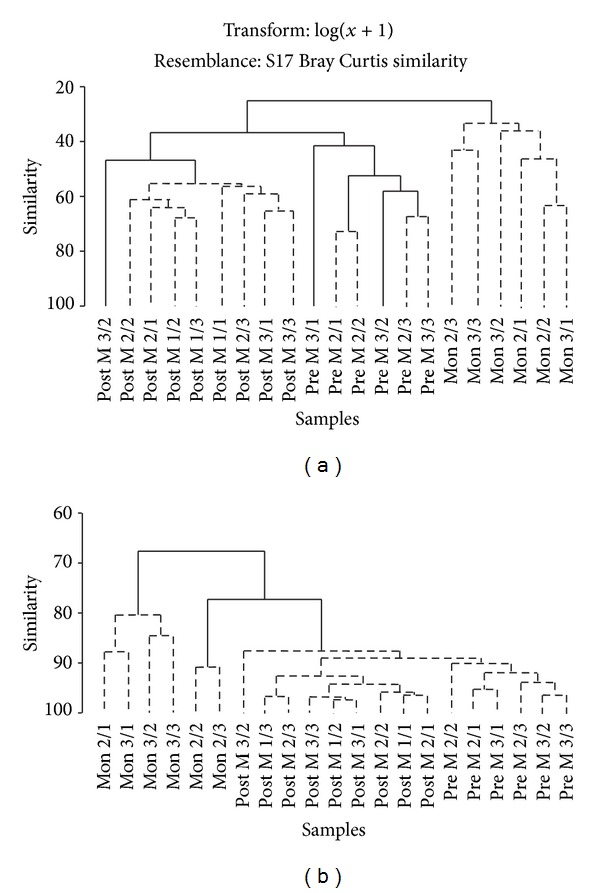
SIMPROF test on dendrogram for (a) abundance and (b) feeding guild from standard hierarchical clustering. Dashed line indicates groups of samples not separated (*P* < 0.05).

The SIMPROF test detected that the Pre M cluster showed two significant subgroups (Figure 3(a)). The Stns 2/1 and 2/2 were 73% similar, and sixteen taxa contributed to 77% of the similarity. On the basis of percentage contribution, the important taxa were* Mediomastus* sp., *Cyathura *sp., *Nephtys polybranchia* and *Tharyx* sp. Stns 2/3 and 3/3 clustered with 67% similarity and fourteen taxa contributed to the 69% of the overall average similarity. *Mediomastus* sp. (67%),* Tharyx* sp., *Cyathura *sp., *Gibberula* sp., and Nematoda were the most important taxa. The Pre M clusters showed 49% dissimilarity, and 19 taxa contributed significantly to discriminate the two groups (DSIM/SD ratio > 2). The most reliable discriminating species were *Aricidea catherinae*, *Armandia lanceolata*, *Polydora coeca*, *Glycera alba*, and *Synchelidium* sp. Overall, although the abundance of *Mediomastus *sp. was reduced from Pre M to Mon, it was the dominant species both during Pre M (50%; 6, 392 ± 4, 353) and Mon (34%; 1, 488 ± 1, 647). *Cyathura* sp. abundance showed significant seasonal variation (Table 4). The low abundance and species number at Stn 3/1 and 3/2 separated it from rest of the stations. Cluster analysis with SIMPROF tests determined whether there was any significant grouping in the cluster. Therefore, although the Pre M showed clustering in nMDS, SIMPROF was able to detect the variability, and the null hypothesis was rejected indicating variability in the macrofaunal community during this period. 

On the other hand, the null hypothesis was retained for Mon data as all the stations grouped and SIMPROF analyses detected no further structure. Species such as *Mediomastus* sp., *P. pinnata,* and *Aricidea catherinae* (SIM/SD > 2) contributed significantly to characterize the Mon community. The abundance of *P. pinnata* showed significant seasonal variation (Table 4). 

Macrofaunal biomass followed a pattern that was similar to that of the abundance and showed significant seasonal variation (*F* = 8.81; *P* < 0.001). Post hoc test detected Post M biomass to be significantly higher and ranged from 1.93 to 15.56 g m^−2^. Polychaeta was the dominant group during Post M (80%; Avg. 12.54 g m^−2^). However, the biomass was reduced to 3.14 g m^−2^ (37%) and 0.66 g m^−2^ (34%) during Pre M and Mon, respectively. Gastropod biomass increased from 0.12 (Post M) to 2.69 g m^−2^ (Pre M). Biomass of bivalves also increased from 1.90 (Post M) to 2.17 g m^−2^ (Pre M). However, there was 50% reduction in the biomass of crustacean during Pre M, indicating a reversing trend compared to other taxa. In fact, the lowest crustacean biomass of (0.10 g m^−2^) was recorded in Mon. Biomass of the other groups increased from postmonsoon (0.37 g m^−2^) to premonsoon (1.70 g m^−2^) and decreased marginally in monsoon (1.02 g m^−2^).

**Figure 4:**
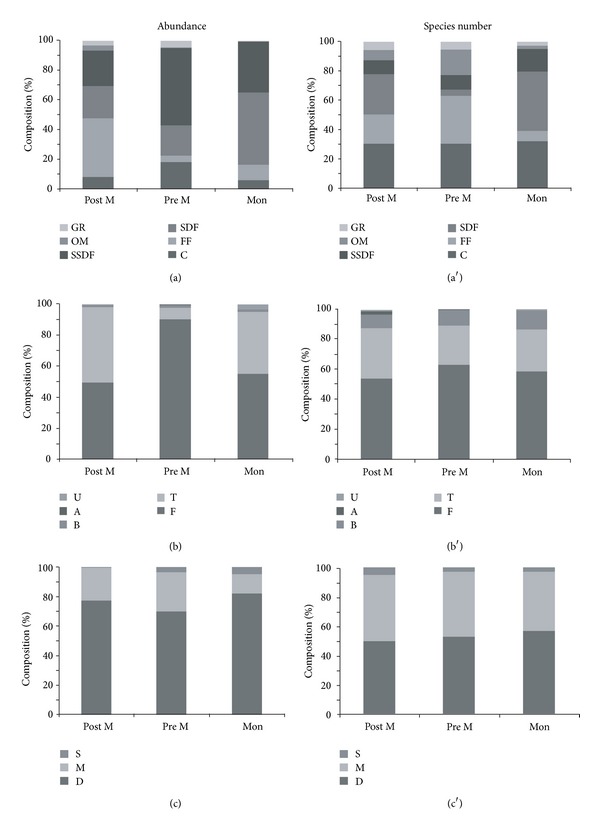
Percent composition of functional groups based on abundance and species number. (a) Feeding guilds, (b) Habit, and (c) Mobility.

The macrobenthic community was dominated by subsurface and surface deposit feeders (36% and 30% Figure 4(a)). SDF increased from Post M to Mon and was the only group present at some stations. SSDF increased from Post M to Pre M and a decline was recorded during Mon. Filter feeders were highest during Post M (20%), and the abundance declined during Pre M (17%) and Mon (7%). Abundance of omnivore (avg. 5%) and grazers (avg. 5%) decreased during Post M, and the lowest values were recorded during Mon season. Two-way ANOVA detected significant seasonal variation in the abundance of C, FF, GR, and OM (Table 5). The result of ANOSIM showed significant seasonal variation in the feeding group (*R* = 0.57; *P* = 0.1%). The SIMPROF showed the clustering of Post and Pre M stations, while Mon stations formed separate cluster (Figure 3(b)). Based on species composition, the community was composed of C (31%), SDF (24%), FF (20%), SSDF (12%), OM (9%), and GR (5%; Figure 4(a′)).

Free-living (49–90%) and tubiculous (7–49%) fauna dominated the macrofauna community and showed significant seasonal variation (Table 5). Burrowing forms contributed about 1-2% (Figure 4(b)) of the total abundance. Habit types based on species composition showed a similar pattern with free-living forms (58%) being the most dominant followed by tubiculous (29%) and burrowers (10%). The other types made up to 3% of the composition (Figure 4(b′)). Most of the macrofaunal species belonged to mobile (39–49%) and discretely mobile (45–52%; Figure 4(c)) forms and showed significant seasonal variation (Table 5). Sessile forms recorded an increase from Post M (6%) to Mon season (9%). Species composition of the discretely mobile forms was dominant at 53% followed by mobile form at 43% (Figure 4(c′)). The macrofaunal community based on habit type (*R* = 0.30; *P* = 2.3%) and motility (*R* = 0.25; *P* = 1.1%) showed significant seasonal variation by two-way ANOSIM. 

Macrofaunal total species abundance showed the best relation with OC, D_50_, sand, mud, and PO_4_ (Table 6). Feeding type showed a similar correlation with environmental variables as observed in total species abundance. Habit type showed significant relation with CHO, D_50_, sand, and PO_4_. The environment variable that best explained the variation of benthic fauna based on the mobility was D_50_. 

## 4. Discussion 

The Kalbadevi Bay with its varying physico-chemical variables provides an opportunity to understand the biodiversity-environment relation of a placer rich tropical coastal habitat. The knowledge of such natural variation in biodiversity pattern will be helpful for effective conservation plans of this ecologically and economically important habitat. The macrofaunal community patterns of Kalbadevi Bay showed that the significant temporal variation was related to changes in the environmental condition (BIO-ENV). The filter feeders dominated along with deposit feeders during the Post M. During the Pre M and Mon periods, the deposit feeders dominated the community. Moreover, the fluctuation observed in the functional diversity pattern of macrofauna was related to the temporal variation in organic matter sedimentation. 

During monsoon, the organic matter increases in the region directly due to riverine transport and indirectly by augmentation of primary production from the nutrients brought from the river runoff [4]. Upwelling during monsoon further enhances the primary production of the region. The end of monsoon is characterized by collapse and sinking of phytoplankton bloom that reaches the benthic system during the Post M period. This sinking organic matter constitutes high-quality food for the benthic fauna. The dominance of sedimentary PRT and high PRT : CHO, LOM : OC, and Sed Phaeo : Sed Chl *a* ratios during Post and Pre M indicates productivity of the region and “newly generated detritus” for the benthic organism [33]. There was no significant seasonal or spatial change in PRT concentration and PRT : CHO ratio. Further, a microcosm study demonstrated the influence of bioavailable iron released from ilmenite by microbial action on the abundance and diversity of phytoplankton [34]. Therefore, it may be possible that in Kalbadevi Bay, iron may not be a limiting factor for phytoplankton as it may be continuously leached from ilmenite (FeTiO_3_). Mats of green algae have been also observed in the sea floor of the bay [34]. This could also explain for the high quality organic matter in the region throughout the year. However, this hypothesis needs to be further studied to confirm the role of iron from ilmenite on the primary production. Therefore, runoff from the adjacent creeks and upwelling coupled with the possible bioavailable iron from ilmenite supports a high primary productivity and quality organic matter for benthos during most part of the year.

The filtering feeding polychaete, unidentified Sabellidae species, was abundant, possibly a response to the good trophic conditions of the water column. The high abundance of Sabellidae species corresponds to the recruitment as the population was dominated by juveniles. There is strong evidence that availability of high quality food is critical for the survival of juveniles and population dynamics of benthic species [35]. *Amplesica* sp., a filtering feeding Amphipod, was also found in high abundance during this period. Although the FF abundance declined during premonsoon, species number showed an increase and was dominated by Bivalvia, indicating a continuous primary production and flux of organic matter in the region. The filter feeders consume the “fresh detritus” from the water column, but sedimented organic matter later in the season is processed by the deposit feeders [36].

The retention of organic matter in sediment is influenced by the particle size [37] which in turn is largely governed by the hydrodynamic of the region. The sediment in the study site was composed of fine sand which has lower holding capacity than mud, resulting in washing out of organic matter. However, the bioturbation activities of macrofauna ensure that the organic matter is transported and stored in deeper sediment for the subsurface deposit feeders. In this study, the bioturbation potential has been inferred from the functional traits. Tube-dwelling surface defecators are considered to have low bioturbation activity, while subsurface depositors and motile forms are good bioturbators [38]. It may also be concluded that the bioturbation activity could be high since the community was dominated by subsurface deposit feeders, free living, burrowing, and mobile forms (mobile and discreetly mobile). The activity of these macrofaunal species possibly helps in the exchange of material not only between the water column and sediment but also to deeper sediment layers. 

Although carnivorous species composition did not fluctuate, abundance was high during Pre M which presumably represents a response to increase in food in terms of surface-deposit feeders. The carnivorous species can facilitate the transport of nutrients retained in the detritivores tissues back in to the mobile pool [39] and hence renew the nutrients for primary producers during the nonmonsoon period. 

The variation in environmental parameters selected species with particular functional trait in which this attribute was consequently reflected in the ecosystem functioning of Kalbadevi Bay. The filter feeders processed organic matter from the water column, while deposit feeders utilized the sedimented detritus. Moreover, the bioturbation activities of macrofauna transported organic matter to deeper sediment for SSDF. The presence of carnivorous species further helped to transfer the nutrients retained in the deposit feeders back into the mobile pool. Thus, the functional diverse macrobenthic community rapidly consumed the organic matter and converted it to benthic biomass which forms the food for organism at the higher trophic level such as the demersal fish. Therefore, niche partitioning by different functional groups resulted in more efficient utilization of organic matter in the Kalbadevi Bay. The results of the present study support our hypothesis that the environmental variation influenced by the annual monsoon influenced the functional diversity pattern of macrofaunal community. Further, the spatial variability of macrofaunal functional diversity pattern was insignificant when compared to the temporal variation. Other studies have also underlined the functional diversity-environmental gradient relationship and its influence in the ecosystem processes [14, 40]. Moreover, several studies have predicted that functional diversity approach is beneficial to determine anthropogenic disturbance [41]. Paganelli et al. [41] observed that the macrofaunal functional diversity pattern increased towards the less impacted sites along the Po River delta and suggested that the riverine input was detrimental for the ecosystem functioning. 

## 5. Conclusion

The present study provides an evaluation of the current condition of Kalbadevi Bay, a potential placer mining site along the central west coast of India. The functional diversity of macrofaunal community showed a temporal variation. The observed variation was related to fluctuations in organic matter and sediment texture driven by seasonal variation in river runoff. The present study is small, but the first step towards understanding the biodiversity-environment relation and the ecological process of potential placer mining sites. An important output is the understanding of the natural variation in the macrofauna functional diversity patterns of an economically significant placer rich bay which could be valuable when mining commences.

## Figures and Tables

**Table 1:** Environmental parameters of Kalbadevi Bay.

	Post M	Pre M	Monsoon
W Chl *a* (*μ*g L^−1^)	0.02–1.2	1.0–1.67	0.28–0.68
W Phaeo (*μ*g L^−1^)	0–0.1	0	0–0.39
Sed Chl *a* (*μ*g g^−1^)	0.1–0.59	0.15–0.7	0.08–0.28
Sed Phaeo (*μ*g g^−1^)	0.3–1.7	0.1–0.45	0–0.48
Sed Phaeo: Sed Chl *a *	1–6	0.4–0.8	0–1.5
LIP (*μ*g g^−1^)	29–584	133–231	19–236
PRT (*μ*g g^−1^)	374–2478	943–3194	203–4640
CHO (*μ*g g^−1^)	132–584	183–317	134–564
PRT: CHO	1–7	3–8	1.5–8
*TSM (mg L^−1^)	0.5–3.6	—	0.4–5
*DO (mg L^−1^)	2.8–3.7	2–3.6	0.3–4.97
* PO_4_ (*μ* mol L^−1^)	0–0.14	0.13–0.22	0.09–0.44
*NO_2_ (*μ* mol L^−1^)	0.08–0.26	0–0.04	0–0.47
*NO_3_ (*μ* mol L^−1^)	0.2–3.27	0.24–0.8	0.24–4.4
*SiO_4 _( mol L^−1^)	0.57–1.76	0.1–1.46	0–1.43

*Source: Anon 2007 [24].

**Table 2:** Coefficients in the linear combinations of variables making up PCs.

Variable	PC1	PC2	PC3
Water parameters			
DO	−0.20	0.07	−0.26
PO_4_	0.15	**−0.35**	**0.31**
NO_2_	0.10	**0.46**	−0.11
NO_3_	0.19	0.01	−0.10
SiO_4_	0.09	0.29	−0.02
W Chl *a *	**−0.29**	−0.21	0.20
W Phaeo	0.21	0.10	−0.20
Sediment parameters			
PRT	**−0.30**	−0.28	−0.13
CHO	0.01	−0.02	**−0.40**
LIP	0.06	−0.05	0.24
LOM	−0.29	−0.28	−0.18
OC	**−0.31**	0.08	−0.22
Sed Chl *a *	−0.26	**−0.33**	−0.04
Sed Phaeo	−0.22	0.09	**−0.43**
Sand	**0.34**	−0.26	−0.19
Mud	**−0.34**	0.26	0.19
D_50_	**0.31**	−0.24	−0.11
Sorting	0.19	−0.22	**−0.39**

Eigen values	6.08	2.63	2.34
% variation	33.8	14.6	13
Cum.% variation	33.8	48.4	61.4

**Table 3:** Result of the two-way ANOVA. Only the variables that were significant are represented. Season/transect showing significantly highest mean values from the SNK test are shown. SNK test Student-Newman-Keuls tests.

Variables	Source of variation	df	MS	*F*	*P *	SNK test
PO_4_	Season	2	0.067	7.82	0.006	Mon
NO_2_	Season	2	0.03	9.73	0.003	Post M
W Chl *a *	Season	2	0.91	15.18	0.0005	Pre M
Sand	Season	2	0.9	15	0.0004	Mon
	Transect	1	1.0	17	0.001	Transect 3
Mud	Season	2	0.93	15.49	0.0004	Pre M
	Transect	1	1.01294	16.91	0.001	Transect 2
D_50_	Season	2	2192.2	6.34	0.013	Mon
	Transect	1	1722.6	4.98	0.045	Transect 3
Sed Chl *a *	Season	2	0.19	6.44	0.012	Pre M
Sed Phaeo	Season	2	0.39	4.96	0.026	Post M
OC	Season	2	0.156	31.36	0.000017	Post M, Pre Mon

**Table 4:** Result of the two-way ANOVA. Only the variables that were significant are represented. Season/transect showing significantly highest mean values from the SNK test are shown. SNK test Student-Newman-Keuls tests.

Variables	Source of variation	df	MS	*F*	*P *	SNK test
Abundance	Season	2	34366.5	27.86	1.77*E* − 07	Post M
Biomass	Season	2	5.68	8.81	9.76*E* − 04	Pre M
Sp. number	Season	2	1401.46	31.65	5.11*E* − 08	Post M, Pre M
Sp. richness	Season	2	43.06	35.63	1.54*E* − 08	Pre M, Post M
Sp. diversity	Season	2	4.7	4.411	2.69*E* − 09	Post M
Unidentified Sabellidae	Season	2	198.49	57.90	7.47*E* − 11	Post M
*Cyathura* sp.	Season	2	74.57	20.67	2.63*E* − 06	Pre M
*P. pinnata *	Season	2	91.94	34.19	2.35*E* − 08	Mon
*Ampelisca *sp.	Season	2	105.46	36.89	1.08*E* − 08	Post M
*Synchelidium *sp.	Season	2	100.62	71.42	6.24*E* − 12	Post M

**Table 5:** Result of the two-way ANOVA. Only the variables that were significant are represented. Season/transect showing significantly highest mean values from the SNK test are shown. SNK test Student-Newman-Keuls tests.

Variables	Source of variation	df	MS	*F*	*P *	SNK test
Feeding guilds						
Carnivores	Season	2	38.53	26.28	2.54*E* − 07	Pre M, Post M
Filter feeders	Season	2	125.28	37.06	7.83*E* − 09	Post M
Omnivores	Season	2	51.35	10.56	3.37*E* − 04	Post M
Grazers	Season	2	91.49	24.23	5.44*E* − 07	Post M, Pre M
Mobility						
Discretely mobile	Season	2	15.79	8.03	1.64*E* − 03	Post M, Pre M
Mobile	Season	2	49.35	19.59	3.60*E* − 06	Post M, Pre M
Habit type						
Free living	Season	2	21.18	13.28	7.38*E* − 05	Pre M, Post M
Tuberculous	Season	2	21.97	10.58	3.32*E* − 04	Post M

**Table 6:** Result of BIOENV analysis.

Variables	Environmental parameters	Global *R*	*P* %
Total abundance	OC, D_50_, Sand, Mud, PO_4_	0.57	1
Feeding guilds	OC, D_50_, Sand, PO_4_	0.69	1
Habit type	CHO, D_50_, Sand, PO_4_	0.43	5
Mobility	D_50_	0.63	1

**Table 7:** Macrofauna from Kalbadevi Bay.

Taxa	Abundance	Feeding	Motility	Habit
**Anthozoa**				
*Virgularia* sp.	25–2,475	FF	S	U
**Hydrozoa**				
*Obelia* colony	25–50	FF	S	A
**Nematoda**	25–1,875	SDF	M	F
**Nemertina**	25–700	C	M	F
**Polychaeta**				
*Ancistrosyllis* sp.	0–25	C	M	F
*Microphthalmus *sp.	0–1,100	GR	M	F
*Hesione* sp.	0–50	O	M	F
*Exogone* sp.	25–75	GR	M	F
*Odontosyllis* sp.	0–50	C	M	F
*Nereis *sp.	25–75	O	D	T
*Perinereis *sp.	0–25	O	D	T
*Eteone* sp.	50–1,025	O	M	F
*Phyllodoce *sp.	50–650	O	M	F
*Phyllodoce castanea *	25–75	O	M	F
*Genetyllis gracilis *	25–475	O	M	F
*Lumbrineris bifilaris *	75–625	C	M	F
*Lumbrineris impatiens *	25–150	C	M	F
*Lumbrineris latreilli *	25–350	C	M	F
*Lumbrineris* sp.	50–150	C	M	F
*Glycera alba *	25–250	C	M	F
*Glycera longipinnis *	25–100	C	M	F
*Glycera prashadi *	250–325	C	M	F
*Glycinde oligodon *	25–175	C	M	F
*Goniadides* sp.	0–25	C	M	F
*Cossura* sp.	25–50	SSDF	M	F
Cirratulidae	75–4,775	SDF	D	F
*Tharyx *sp.	25–2,050	SDF	D	F
*Nephtys* sp.	25–125	C	M	F
*Nephtys polybranchia *	25–425	C	M	F
*Nephtys oligobranchia *	0–125	C	M	F
*Magelona* sp.	25–1,000	SDF	D	F
*Aricidea* sp.	25–225	SDF	D	B
*Aricidea catherinae *	25–250	SDF	D	B
*Levinsenia* sp.	25–50	SDF	D	B
*Protodorvillea egena *	25–550	C	M	F
*Lysidice* sp.	25–675	C	S	B
*Diopatra claparedii *	25–75	C	D	T
*Diopatra *sp.	0–25	C	D	T
*Onuphis holobranchiata *	0–25	C	D	T
*Onuphis eremita *	25–175	C	D	T
*Eunice indica *	25–300	C	NA	NA
*Orbinia *sp.	0–25	SSDF	M	F
*Scoloplos marsupialis *	25–100	SSDF	M	F
*Scoloplos* sp.	25–100	SSDF	M	F
*Paraprionospio pinnata *	25–3,350	SDF	D	T
*Prionospio* sp.	25–225	SDF	D	T
*Minuspio cirrifera *	25–625	SDF	D	T
*Dipolydora coeca *	25–1,100	SDF	D	T
*Pseudopolydora* sp.	50–5,675	SDF	D	T
*Capitella minima *	25–425	SSDF	D	T
*Mediomastus *sp.	225–15,200	SSDF	D	F
*Euclymene insecta *	25–100	SSDF	D	T
*Axiothella* sp.	25–200	SSDF	D	T
*Armandia lanceolata *	25–325	SSDF	D	F
Flabelligeridae	0–25	SDF	D	F
*Poecilochaetus serpens *	25–100	SDF	D	F
*Owenia* sp.	0–25	SDF	S	T
*Trochochaeta orissae *	0–25	SDF	S	T
*Phyllochaetopterus socialis *	25–100	SDF	D	T
*Sternaspis scutata *	25–525	SSDF	S	B
Sabellidae	25–16,925	FF	D	T
Terebellidae	25–1,125	SDF	D	T
*Terebellides* sp.	25–175	SDF	S	T
*Terebellides stroemii *	0–25	SDF	D	T
*Amage *sp.	25–75	SDF	M	T
**Oligochaeta**	0–25	SSDF	D	T
**Sipuncula**	0–25	SDF	S	F
**Phoronida**	25–50	FF	D	T
**Bivalvia**				
*Cardites bicolor *	0–25	FF	D	B
*Cuspidaria *sp.	25–75	C	D	F
*Venerupis *sp.	0–50	FF	D	F
*Donax *sp.	25–50	FF	D	B
*Modiolus *sp.	25–275	FF	D	A
*Timoclea scabra *	25–1,150	FF	D	F
*Mactra* sp.	0–200	FF	D	F
*Tellina ala *	25–150	FF	D	F
*Tellina *sp.	25–1,175	FF	D	F
*Solen *sp.	0–25	FF	D	B
**Gastropoda**				
*Babylonia spirata *	0–50	C	M	NA
*Bittium* sp.	0–25	SDF	M	F
*Umbonium vestiarium *	0–25	FF	M	F
*Surcula* sp.	0–25	C	NA	F
*Turritella* sp.	0–25	C	D	B
*Calyptraea* sp.	0–25	FF	M	C
*Cylichna *sp.	25–100	C	M	F
*Oliva* sp.	25–50	C	M	F
Mitridae	0–25	C	NA	F
*Cypraea* sp.	0–25	C	M	NA
*Polinices* sp.	0–25	C	M	F
**Cumacea**				
Bodotriidae	25–1,100	GR/DR	M	F
*Cyclaspis* sp.	25–475	GR/DR	M	F
**Isopoda**				
*Cyathura* sp.	25–1,375	C	M	F
**Tanaidacea**	100–300	SDF	D	T
**Amphipoda**				
*Ampelisca* sp.	25–2,300	FF	M	T
*Synchelidium* sp.	25–475	C	M	F
Haustoriidae	25–75	C	M	B
*Ampithoe *sp.	25–100	BR	D	B
*Photis* sp.	25–400	FF	D	T
Isaeidae	50–1,000	FF	D	T
Melphidippidae	75–100	FF	D	F
Corophiidae	0–25	FF	D	T
*Gammarus* sp.	0–25	FF	NA	T
Liljeborgiidae	50–75	SDF	M	NA
Stegocephalidae	0–25	C	M	F
Phoxocephalidae	0–25	C	M	B
Caprellidae	25–175	GR	M	F
**Decapoda**				
Brachyura	0–25	NA	NA	NA
Penaeidae	0–25	NA	NA	NA
*Pagurus *sp.	0–25	O	M	F
**Ostracoda**	25–1,075	NA	NA	NA
**Ophiuroidea **	25–150	NA	NA	NA
**Amphioxiformes**				
*Branchiostoma lanceolatum *	0–25	FF	NA	B

NA: not available.

## Appendix

For more details see Table 7.
